# *Salmonella enterica* Serotype Typhi Bacteremia Complicating Pregnancy in the Third Trimester

**DOI:** 10.1155/2017/4018096

**Published:** 2017-01-19

**Authors:** George F. Guirguis, Krunal Patel, Lisa Gittens-Williams, Joseph J. Apuzzio, Kristina Martimucci, Shauna F. Williams

**Affiliations:** ^1^Division of Maternal Fetal Medicine, Department of Obstetrics, Gynecology and Women's Health, Rutgers New Jersey Medical School, Newark, NJ, USA; ^2^Division of Maternal Fetal Medicine & Surgery, Department of Obstetrics, Gynecology and Women's Health, Hackensack University Medical Center, Hackensack, NJ, USA

## Abstract

*Background*.* Salmonella enterica* serotype Typhi (*S.* Typhi) is an anaerobic gram-negative enteric rod that causes infection when contaminated food or water is ingested and may cause illness in pregnancy.* Case*. This is a patient who presented at 31 weeks' gestation with abdominal pain and fever and was diagnosed with* S. *Typhi bacteremia.* Conclusion*.* S*. Typhi should be considered in febrile patients with recent travel presenting with abdominal discomfort with or without elevated liver enzymes.

## 1. Introduction

There are approximately 5,700 cases of typhoid fever in the United States each year; approximately 75% are associated with international travel. Because of advances in food handling and sanitation, rates of* Salmonella enterica* serotype Typhi (*S*. Typhi) infection have decreased significantly in the United States; however it is reported in over 20 million people each year in developing countries [1]. Patients infected with* S*. Typhi may experience complications that are local to the gastrointestinal tract or systemic. Patients with severe infection with a heavy bacterial load may present with septic shock, acute renal failure, hepatic dysfunction [2, 3], myocardial failure [4], or disseminated intravascular coagulation [5]. In 15% to 25% of cases, patients may have leukopenia and neutropenia [6]. The development of acute respiratory distress syndrome (ARDS), gastrointestinal perforations, and development of neurological complications, such as encephalopathy, are rare but serious complications of typhoid fever [7].

When infection occurs during pregnancy, consequences can be significant to both the mother and fetus such as pregnancy loss, intrauterine fetal demise, and preterm labor [8, 9].* S. *Typhi can enter the maternal bloodstream and cross the placenta. Vertical transmission of* S. *Typhi occurs via transplacental spread of the organism and neonatal infection can also occur by transmission through the lower birth canal or from exposure to maternal blood [10]. The manifestation of* S. *Typhi may vary; however abdominal pain, fever, nausea, and vomiting are usually present. In pregnancy, this combination of symptoms presents a diagnostic challenge, as the differential diagnosis of abdominal pain is long. Here, we report a case of a patient of* S.* Typhi who presented with symptoms consistent with clinical chorioamnionitis.

## 2. Case Presentation

A 26-year-old patient, gravida 1 para 0, at 31 weeks and 6 days' gestation presented to the labor and delivery suite with complaints of lower abdominal pain for two days associated with nausea and several episodes of vomiting. She had immigrated to the United States from Nigeria two weeks prior to presentation. During the first trimester of pregnancy, she was diagnosed with malaria and treated with an antimalarial; she did not recall the specific treatment regimen. She denied other significant past medical or surgical history.

On examination, the patient had a temperature of 38.8°C, pulse of 126 beats/min, and blood pressure of 101/60 mm Hg. Abdominal examination revealed tenderness of the uterine fundus as well as costovertebral angle tenderness. Fetal monitoring was significant for fetal tachycardia of 160 beats/min, and there were no contractions on the tocodynamometer. Further testing upon admission included a urinalysis and urine culture, blood cultures, complete blood count, comprehensive metabolic panel, malaria smear, and an in vitro immunochromatography assay for the qualitative detection of plasmodium antigens. The patient was given ceftriaxone and acetaminophen for a presumptive diagnosis of pyelonephritis. Laboratory results showed white blood cell count of 9.1/L, hemoglobin of 10.1 g/dL, platelet count of 382/L, neutrophil count of 76%, aspartate aminotransferase of 68 IU/L, and alanine aminotransferase of 39 IU/L. A urinalysis showed no white blood cells and nitrites and leukocyte esterase were negative. Maternal blood PCR testing for malaria was negative.

Differential diagnosis included chorioamnionitis, pyelonephritis, preterm labor, pneumonia, urinary tract infection, cystitis, appendicitis, and ovarian torsion. A diagnosis of chorioamnionitis was made based on the clinical criteria of fetal tachycardia, uterine tenderness, and maternal fever. Betamethasone was given for fetal lung maturity and magnesium sulfate was administered for neuroprotection. Preliminary blood culture results were positive for gram-negative rods in two anaerobic bottles. The antibiotic regimen was altered for broader coverage to meropenem and ampicillin. Labor induction with misoprostol was initiated. During the induction, fetal heart tracing showed fetal tachycardia accompanied by minimal variability and a cesarean delivery was performed because of concern for the fetal status. A live female infant with a birth weight of 1570 grams was delivered with Apgar scores of 8 and 9 at 1 and 5 minutes, respectively. Final blood culture results showed* S. *Typhi on postoperative day 1. The regimen was changed to ceftriaxone and clindamycin once sensitivities were available and continued for 72 hours. Repeat blood and stool cultures after antibiotic initiation were negative for* S.* Typhi. Placental pathology was negative for histologic chorioamnionitis.

The patient was discharged on postoperative day 5 in stable condition on oral levofloxacin for an additional 7 days. The neonate was admitted to the intensive care unit after delivery, and blood cultures were negative. The neonate was discharged home on day 29 of life.

## 3. Discussion

*S. *Typhi is an anaerobic gram-negative enteric rod that has the potential to cause infection when contaminated food or water is ingested. The most common symptoms and findings are generalized malaise, fever, abdominal discomfort, diarrhea, and headache. Patients may also have constipation, splenomegaly, anorexia, chills, bradycardia, and leukopenia [11]. Rose spots are a pink macular rash that can be seen in up to 30% of patients, but, presenting early in the course, they can be missed [7]. In pregnancy, hemolysis with fever and hepatic dysfunction has been reported [8]. Pregnancy complications also include second trimester loss [9] and preterm delivery [12].* S*. Typhi should be included in the differential in pregnant patients who present with abdominal pain and fever after travel to endemic areas. Additionally, other infectious diseases should be considered including malaria, dengue, leishmaniasis, and leptospirosis [13].

Clinical chorioamnionitis is diagnosed with two or more of the following: fever of 100.4 F, uterine tenderness, maternal or fetal tachycardia, or amniotic fluid that is foul smelling or purulent in appearance. Following a diagnosis of clinical chorioamnionitis, broad spectrum antibiotics are administered and delivery is recommended [14]. In this case, the patient presented with uterine tenderness, fetal tachycardia, and fever, so she was diagnosed with clinical chorioamnionitis and induction of labor was initiated. Because of the neonatal complications associated with chorioamnionitis, including early onset neonatal sepsis, pneumonia, intraventricular hemorrhage (IVH), and long-term neurological disability, awaiting blood or stool culture results poses a significant dilemma.

For a pregnant patient with* S.* Typhi, once delivered, the neonate should be monitored for signs of* S*. Typhi. Lethargy, respiratory distress, sepsis, icterus, and pneumonia can indicate neonatal infection and may occur up to 10 days after birth [15].

The recommended antibiotic regimen for* S. *Typhi infection in pregnancy is a third-generation cephalosporin or azithromycin. In the nonpregnant patient, if susceptible, fluoroquinolones such as ciprofloxacin may be given [11]. Fluoroquinolones could be considered for use postpartum in patients who are not breastfeeding. Treatment is critical to decrease the risk of complications and decrease relapse and carriage rates. If the patient develops chronic carriage of* S*. Typhi, then an antibiotic course of 4–6 weeks is recommended [11].

Patients who travel to endemic areas during pregnancy should be aware of the risk of acquiring* S. *Typhi. To minimize this risk while traveling, patients should practice strict food hygiene and avoid uncooked foods. Two vaccines are available, a live attenuated vaccine (Ty21a) and the Vi capsular polysaccharide vaccine. As the Ty21a is a live vaccine, the Vi vaccine is preferred if needed in pregnancy [16]. Patients may also contract infections in their own homes as a result of contact with family members who are chronic carriers. Thorough cooking of poultry and eggs and care when handling household pets with thorough hand washing are recommended.

Given the recent concern in the modern era of travel of widespread epidemics of various microorganisms, clinicians should always ask their patients about travel and continue to have a high index of suspicion for infections that are endemic to the countries traveled.
